# Sustainability-induced loyalty in festival tourism: Examining the mediating pathways between sustainable practices and visitor behavioral support

**DOI:** 10.1371/journal.pone.0348506

**Published:** 2026-05-14

**Authors:** Yuanxiang Peng

**Affiliations:** Department of Tourism Management, International School, Beijing Youth Politics College, Beijing, China; Zhejiang Shuren University, CHINA

## Abstract

This research constructs a comprehensive theoretical framework examining the multidimensional influence mechanisms of music festival sustainability practices (environmental, social, economic dimensions) on visitor behavioral intentions. Based on the integration of sustainable tourism, environmental awareness, perceived value, and tourist satisfaction theories, the study proposes a hypothetical model encompassing both direct and mediating effects. Through a survey of 500 participants at a major international music festival, advanced statistical techniques validate the complex mechanisms through which sustainability practices influence visitor behavioral intentions via multiple pathways. Findings show: (1) all three dimensions of sustainability practices exert significant direct positive effects on behavioral intentions, with environmental sustainability having the strongest influence (β = 0.187, p < 0.001); (2) environmental awareness, perceived value, and tourist satisfaction play significant mediating roles between sustainability practices and behavioral intentions; (3) sustainability practices influence behavioral intentions through sequential mediating pathways of “environmental awareness to perceived value” and “perceived value to satisfaction.” These findings suggest that sustainability practices should be viewed as strategic assets enhancing visitor experiences rather than merely compliance obligations. By refining sequential mediations in a music festival context (extending linear models like VBN), the study addresses a key “black box” in pro-environmental behavior, while providing empirical guidance for organizers to build integrated strategies that promote resilient tourism amid post-COVID recovery and overtourism pressures.

## 1. Introduction

Music festivals have emerged as pivotal elements of destination development strategies, transcending mere entertainment functions to become complex socio-economic catalysts [1,2]. These temporally bounded celebrations constitute what Getz conceptualizes as “planned events,” characterized by “spatial and temporal uniqueness, stemming from interactions among setting, people, and management systems” [1]. Contemporary event tourism discourse has shifted from purely economic impact assessments to holistic evaluations incorporating social and environmental dimensions, driven by heightened stakeholder awareness of sustainability amid global climate challenges [3,4].

Music festivals, with their resource-intensive production requirements and concentrated ecological impacts, face particular scrutiny regarding sustainability credentials [3]. As Laing asserts, “the challenge of balancing economic viability with environmental responsibility and social inclusivity represents the central problematic confronting contemporary event organizers” [5]. Recent developments, including greening initiatives at Western festivals like Coachella and Glastonbury, as well as non-Western events like the Rainforest World Music Festival, underscore the urgency of sustainable practices amid climate crises, post-COVID recovery, and risks of greenwashing [6].

Despite the growing implementation of sustainability practices across the festival sector, a significant knowledge gap exists regarding how these initiatives influence visitor perceptions, evaluations, and behavioral intentions [7,8]. This deficit is particularly pronounced concerning the psychological mechanisms through which sustainability practices translate into visitor responses [9]. Key gaps include the undertheorized integration of multidimensional sustainability (environmental, social, economic) in event tourism [10]; reliance on binary assessments rather than visitor perceptions [7]; disconnects between theory and empirical pathways to behavior [8]; and Western-centric biases that limit model generalizability, though emerging non-Western studies (e.g., [11]) begin to mitigate this.

To address these gaps, this study develops and validates a structural equation model examining music festival sustainability practices’ impact on visitor behavioral intentions, with objectives to: assess direct effects of environmental, social, and economic dimensions; investigate mediating roles of environmental awareness, perceived value, and satisfaction; analyze sequential mediations; and integrate a multidimensional framework elucidating influence mechanisms.

This study specifically extends the Value-Belief-Norm (VBN) theory and Model of Goal-directed Behavior (MGB) by testing their application in festival contexts, where sequential paths refine the linear processes from values to behaviors. This research innovates by: adopting a multidimensional sustainability approach beyond environmental focus [12]; employing SEM for simultaneous pathway analysis [13]; identifying practical mechanisms for behavior influence [14]; and refining the “black box” via sequential mediations, uniquely extending VBN and MGB in immersive festival settings—contrasting with linear applications in general tourism(e.g., [15]).

This study employs structural equation modeling (SEM) to examine complex relationships between latent constructs through simultaneous estimation of multiple dependency relationships [13,16]. The conceptual model posits that music festival sustainability practices influence visitor behavioral intentions both directly and indirectly through multiple mediating pathways, integrating three primary theoretical perspectives: value perception theory, environmental consciousness theory, and satisfaction theory [17]. Analysis follows SEM best practices, including measurement validation and structural testing, with robustness checks for bias (e.g., via latent method factor) and multi-group comparisons (detailed in Methodology).

## 2. Literature review and research hypotheses

### 2.1. Current research status of music festival tourism

Music festival tourism scholarship has evolved substantially over three decades, transitioning from descriptive case studies to sophisticated theoretical frameworks. Getz established foundational conceptual architecture, defining music festivals as planned events with distinctive experiential characteristics [1]. Early management-oriented research utilized case methodologies [4], while recent approaches have deployed advanced analytical frameworks—exemplified by Chang et al.’s Strategic Issue Analysis-Network Map for urban festivals [4]—though construct definition inconsistencies persist across studies.

Theoretical discourse has expanded beyond managerial perspectives to encompass experiential dimensions. Matheson explored relationships between music, emotional response, and authenticity perceptions [18], while Neuhofer et al. conceptualized festivals as liminal spaces facilitating identity formation [19]. Digital integration trends, including Montoro-Pons et al.’s online search pattern analysis [20], complement post-COVID adaptations (e.g., hybrid formats), though non-Western contexts remain underexplored [21].

Stakeholder management approaches have demonstrated increasing sophistication. Van Niekerk advanced beyond descriptive identification to prescriptive engagement strategies through stakeholder matrix applications [22]. Behavioral outcome research positioning visitor intentions as dependent variables represents significant recent development. However, methodological heterogeneity hinders synthesis, Western bias limits generalizability [10], and construct operationalizations vary substantially, as evidenced by contrasting African festival studies [21]. Table 1 synthesizes representative works, highlighting gaps this study addresses through sequential mediation modeling in diverse contexts.

**Table 1:** Summary of Key Literature on Music Festival Tourism.

Study	Key Findings	Methods	Limitations	Relevance to This Study
Getz [1]	Defined event tourism foundations	Conceptual review	Lacks empirical testing	Basis for event uniqueness in sustainability model
Chang et al. [4]	Hierarchical factors in urban festivals	SIA-NRM analysis	Urban-focused	Extends to sustainability hierarchies
Neuhofer et al. [19]	Transformative experiences	Qualitative	Western bias	Links to perceived value mediation
Montoro-Pons et al. [20]	Online search patterns	Quantitative analysis	Digital only	Informs behavioral intentions paths

### 2.2 Sustainable development and event tourism

Sustainability integration into event tourism discourse represents significant paradigmatic reconfiguration. Dickson et al.’s comprehensive critical review established foundational knowledge architecture for environmentally sustainable events, identifying conceptual frameworks, methodological approaches, and empirical findings [23]. Definitional evolution has progressed from environmental focus toward multidimensional conceptualizations. Jones advocated incorporating social and economic aspects [3], yet empirical studies often prioritize environmental dimensions, producing imbalanced frameworks.

Environmental dimensions have dominated scholarly attention. Laing et al. explored challenges implementing environmental initiatives, identifying organizational, financial, and logistical barriers [24]. Waste management emerged as operational priority, exemplified by Martinho et al.’s case study of Portugal's Andanças Festival [25]. Methodological advancements include Collins et al.’s Ecological Footprint application for festival impact measurement [9]. However, environmental dominance overlooks synergies with social/economic dimensions, as recent mediation studies critique [26].

Sustainability integration into festival design has received increasing attention. Wong et al. examined relationships between green event attributes, value perceptions, and consumer involvement [8]. Limitations include environmental bias, heterogeneous methodologies hindering comparisons, and understudied visitor perceptions—gaps this multidimensional mediation model addresses by linking sustainability to behavioral outcomes.

### 2.3. Research on tourist environmental awareness and behavioral intentions

Tourist environmental awareness and behavioral intentions scholarship has produced sophisticated theoretical explanations for pro-environmental behaviors. Theoretical evolution progressed from Lück's New Environmental Paradigm adaptation for tourism [27] to Üzülmez et al.’s domain-specific environmental consciousness distinctions [28]. Value-Belief-Norm (VBN) theory links values to behaviors [15], though festival-specific nuances remain underexplored.

Behavioral dimensions have gained scholarly prominence. Sharma and Gupta extended VBN theory to national park visitors in emerging economies, establishing theoretical linkages between values, environmental beliefs, personal norms, and pro-environmental behaviors [15]. Festival-specific research reveals unique characteristics, including Alonso-Vazquez et al.’s examination of eco-friendly behaviors at Australian folk festivals [29]. Recent studies have extended moderated mediations in sustainable loyalty, bridging gaps in non-Western settings [30,31].

Paudel employed extended Model of Goal-directed Behavior (MGB) combined with New Ecological Paradigm scale to investigate trekkers’ environmentally friendly behaviors [31]. Song et al. examined how environmental perceptions influence festival visitor decision-making through extended MGB, providing theoretical links between perceptions and behavioral intentions [7].

Economic dimensions of environmental awareness have been explored through willingness-to-pay research. Myung investigated relationships between environmental knowledge, attitudes, and premium pricing willingness for environmentally friendly experiences [32]. Event contexts’ importance in understanding environmental awareness emerged as significant research stream. Mair et al. conceptualized sustainability events as behavior change educational interventions influencing attitudes and competence [33].

These streams link awareness to intentions but minimally explore sequential mediations, which this model contributes within festival contexts.

### 2.4. Perceived value theory

Perceived value theoretical conceptualization has evolved from unidimensional economic formulations toward multidimensional frameworks accommodating complex experiential dimensions. Sánchez et al. established foundational multidimensional framework delineating functional, emotional, and social value components, transcending reductionist price-quality conceptualizations [34].

Contextual specificity investigations span diverse tourism domains. Eid and El-Gohary identified distinctive evaluative criteria reflecting cultural and religious specificities in Muslim tourist contexts [35]. Peña et al. investigated perceived value dimensions within rural tourism [36]. Cross-cultural examinations reveal contingencies, yet often underintegrate sustainability dimensions [35,36].

Heritage tourism contexts have generated particularly nuanced understandings. Chen et al. examined interrelationships between experience quality, perceived value, satisfaction, and behavioral intentions among heritage tourists, establishing theoretical linkages between experiential attributes and evaluative judgments [37]. Pandža Bajs investigated tourist perceived value in Dubrovnik, establishing empirical relationships between perceived value, satisfaction, and behavioral intentions [38].

Nature-based tourism contexts have generated distinctive insights. Kim et al. investigated perceived value and flow experience within nature-based settings, establishing theoretical linkages between environmental attributes, psychological states, and evaluative judgments [39]. Co-creation perspectives introduced dynamic value formation conceptualizations. Prebensen et al. examined co-creation and mastering efficacy on perceived value and satisfaction, reconceptualizing tourists as active value creators [40]. Extensions supporting affordability in loyalty align with our mediation hypotheses linking value to sustainable behaviors [26].

### 2.5. Research on tourist satisfaction

Tourist satisfaction scholarship has evolved from transactional to holistic frameworks. Teixeira et al. analyzed relationships between tourist events, satisfaction, and regional tourism competitiveness, conceptualizing satisfaction as transcending individual outcomes to constitute strategic variables in destination competitiveness [41].

Resident and visitor perspectives have emerged as prominent analytical dimensions. Fytopoulou et al. explored dual perspectives of resident perceptions and visitor satisfaction in local development contexts, theoretically linking host community attributes to visitor experiences [42]. Specialized contexts provide valuable domain insights but exhibit limited focus on host impacts in non-Western contexts [21,43]. Saha et al. conducted comparative analysis of experience quality using both Structural Equation Modeling and fuzzy-set Qualitative Comparative Analysis, integrating variable-centered and configuration-centered approaches [44].

Authenticity dimensions have received increasing scholarly attention. Novello et al. examined influence of event authenticity and quality attributes on behavioral intentions, establishing theoretical linkages between perceived authenticity, satisfaction formation, and behavioral responses [45]. Mediation effects have been extensively investigated. Altunel and Erkurt examined mediation effect of tourist experience and satisfaction on relationships between involvement and recommendation intention within cultural tourism, developing process models explicating sequential relationships [46]. These mediation models position satisfaction as key mediator in sustainability-loyalty paths, though festival-specific nuances remain understudied [26].

### 2.6. Research hypotheses development

Building upon the foregoing synthesis, this study constructs integrated theoretical architecture synthesizing environmental psychology, consumer behavior theory, and tourism experience models. The conceptual model posits sustainability practices influence behavioral intentions both directly and indirectly through multiple mediating pathways. By incorporating sequential mediations, this model extends linear models (e.g., Sharma & Gupta, VBN in parks [15]; Song et al., extended MGB in festivals [7]), responding to calls for nuanced sustainable tourism analyses [26] by refining VBN linearity and MGB processes through embedding sequential paths in immersive festival contexts with event-specific variables.

Theoretical relationships between sustainability practices and behavioral intentions stem from well-documented linkages between festival attributes and visitor responses. Son and Lee identified relationships between festival quality dimensions and visitor behavioral intentions, providing empirical basis for hypotheses that organizational practices influence visitor behaviors via perceptual processes [47]. Extending planned behavior theory applications [48], these relationships justify direct effects, though previous models often fail to account for sustainability's multidimensionality—environmental practices may be more visible, thus more closely linked to behavioral intentions [3].

Song et al.identified relationships between environmental perceptions and decision-making variables in extended goal-directed behavior models [7], empirically supporting hypothesized direct effects between environmental sustainability practices and behavioral intentions.

Based on this theoretical foundation, the following direct effect hypotheses are proposed:

H1a: Environmental sustainability practices (ESP) have a positive effect on visitor behavioral intentions (BI)
H1b: Social sustainability practices (SSP) have a positive effect on visitor behavioral intentions (BI)
H1c: Economic sustainability practices (ECP) have a positive effect on visitor behavioral intentions (BI)

Mediating role of environmental awareness derives from established theoretical work. Paudel extended model of goal-directed behavior and new ecological paradigm scale to investigate trekkers’ environmentally friendly behavior, finding environmental awareness mediates between contextual factors and behavioral outcomes [31]. Mair et al. demonstrated how sustainability events promote pro-environmental behaviors by increasing participants’ environmental awareness and action competence [33].

Mediating role of perceived value between experience quality and behavioral intentions has been established in tourism research. Chen et al. demonstrated how perceived value mediates relationships between experience quality dimensions and behavioral intentions [37].

Mediating role of satisfaction between event attributes and behavioral intentions derives from established frameworks. Teixeira et al. demonstrated how satisfaction mediates relationships between event characteristics and behavioral outcomes [41].

Based on these theoretical foundations, the following mediating effect hypotheses are proposed:

H2a-c: Environmental awareness (EA) mediates the relationship between sustainability practices (ESP/SSP/ECP) and behavioral intentions (BI)
H3a-c: Perceived value (PV) mediates the relationship between sustainability practices (ESP/SSP/ECP) and behavioral intentions (BI)
H4a-c: Tourist satisfaction (TS) mediates the relationship between sustainability practices (ESP/SSP/ECP) and behavioral intentions (BI)

Sequential mediation possibilities arise from theoretical propositions about experience evaluation processes. Chen et al. identified sequential relationships between experience quality, perceived value, satisfaction, and behavioral intentions in heritage tourism contexts, demonstrating these variables can be organized into causal chains [37]. These hypotheses enable testing direct, mediated, and sequential effects relationships, providing richer understanding of sustainability's role in festival loyalty during global crises.

Based on these theoretical propositions, the following sequential mediating effect hypotheses are proposed:

H5a-c: There is a significant sequential mediating effect of sustainability practices (ESP/SSP/ECP) → environmental awareness (EA) → perceived value (PV) → behavioral intentions (BI)
H6a-c: There is a significant sequential mediating effect of sustainability practices (ESP/SSP/ECP) → perceived value (PV) → tourist satisfaction (TS) → behavioral intentions (BI)

Combined, these sets of hypotheses constitute an integrated test of direct, mediated, and sequential effects hypotheses that provide rich support for sustainability’s role in festival loyalty during global crises.

## 3. Research methodology

### 3.1. Questionnaire design and measurement scales

The operationalization of theoretical constructs followed established psychometric protocols encompassing construct specification, literature-derived item generation, expert validation, pilot testing, and reliability assessment [49]. This systematic approach ensured construct validity while maintaining contextual relevance to festival environments.

Measurement scales were adapted from validated instruments with modifications addressing festival-specific characteristics. **Environmental sustainability practices (ESP, 3 items)** were operationalized through Lück's New Environmental Paradigm adaptation for tourism contexts [27], modified to capture festival-specific environmental initiatives (e.g., waste management visibility, renewable energy deployment). **Social sustainability practices (SSP, 3 items)** adapted Wong et al.’s green event attributes scale [8], emphasizing community engagement and social inclusivity dimensions. **Economic sustainability practices (ECP, 3 items)** drew from Jones’ multidimensional sustainability framework [3], focusing on local economic integration and fair pricing transparency observable in festival operations.

**Environmental awareness (EA, 3 items)** employed Üzülmez et al.’s domain-specific environmental consciousness scale [28], contextualized for festival settings to capture heightened ecological sensitivity induced by sustainability exposure. **Perceived value (PV, 4 items)** adapted Sánchez et al.’s multidimensional value framework [34], incorporating functional, emotional, and social dimensions with sustainability-enhanced value perceptions. **Tourist satisfaction (TS, 3 items)** utilized Thrane's festival satisfaction scale [50], validated specifically within music festival contexts. **Behavioral intentions (BI, 5 items)** integrated Tkaczynski and Stokes’ FESTPERF instrument [51] with modifications capturing revisit intentions, recommendation behaviors, and willingness-to-pay premiums for sustainable festivals.

Scale selection prioritized psychometric rigor and contextual appropriateness. The differential item allocation (3–5 items per construct) reflected theoretical complexity and measurement precision requirements: PV required four items to capture multidimensional value facets, while BI necessitated five items to encompass diverse behavioral manifestations (revisit, recommend, premium payment, positive word-of-mouth, continued support). All constructs utilized 5-point Likert scales (1 = Strongly Disagree to 5 = Strongly Agree) with balanced wording and appropriate anchoring to mitigate acquiescence bias and response set tendencies.

Methodological safeguards included: (1) expert panel review (n = 5 academics; n = 3 festival practitioners) assessing content validity and item clarity; (2) pilot testing (n = 90) evaluating comprehension and psychometric properties; (3) back-translation procedures for multilingual versions ensuring semantic equivalence; and (4) systematic cognitive interviews (n = 15) identifying interpretation difficulties. Pilot phase reliability assessment demonstrated acceptable internal consistency (Cronbach's α range: 0.84–0.91), justifying progression to full-scale data collection. Complete measurement items, sources, and adaptation rationale are provided in S1 Appendix A.

### 3.2. Sampling method and data collection

The empirical validation employed purposive sampling through systematic intercept procedures at the Emerald Coast Music Festival (ECMF), an annual three-day international event in Southeast Asia with documented attendance exceeding 120,000 visitors. This festival was selected based on: (1) substantial scale ensuring sample heterogeneity; (2) established multidimensional sustainability practices across environmental (solar power, zero-waste programs), social (community engagement, accessibility), and economic dimensions (local vendor support); and (3) ISO 20121 certification confirming verified sustainability implementation rather than symbolic gestures [48].

Trained research assistants stationed at six sampling locations (main entrances, food areas, performance venues) approached every fifth visitor passing predetermined intercept points across the festival's three-day duration. Sampling windows were distributed temporally (Friday 18:00–22:00, Saturday 12:00–18:00, Sunday 14:00–20:00) to capture experiential variations throughout the festival lifecycle. Of 712 individuals approached, 500 completed questionnaires (response rate: 70.2%). Non-participation (n = 212) was attributed to: time constraints (46.2%), language barriers (22.2%), disinterest (17.9%), incomplete festival experience (9.9%), and technical issues (3.8%). Non-response analysis comparing early versus late respondents revealed no significant differences across demographic variables or key constructs, suggesting minimal non-response bias.

Data collection utilized tablet-based self-administered questionnaires with programmed logic checks ensuring response validity. The recruitment period spanned January 15 to March 15, 2024. Participants provided informed consent via digital protocol presented at survey initiation (QR code access), with voluntary participation and anonymity explicitly stated. No minors participated. Consistent with institutional guidelines and Declaration of Helsinki principles, the low-risk anonymous design warranted waiver of written consent, with survey completion constituting implicit consent.

The instrument was developed in three language versions (English, Mandarin Chinese, Bahasa Malaysia) following rigorous back-translation procedures [49]: forward translation by certified translators, independent back-translation, expert panel review for semantic equivalence (κ > 0.85), and pilot testing (n = 30 per language). Pilot analysis confirmed measurement equivalence across versions (Cronbach's α: 0.89–0.92). Research assistants received standardized training emphasizing neutral presentation to minimize interviewer effects. The final sample (N = 500) substantially exceeded minimum requirements for structural equation modeling with adequate statistical power.

This study was exempted from formal ethical approval by Beijing Youth Politics College, as it involved an anonymous, low-risk survey with no collection of personally identifiable information. The exemption was granted on October 20, 2025, in accordance with institutional guidelines and the principles of the Declaration of Helsinki. Participants were provided with informed consent information at the beginning of the tablet-based questionnaire, explaining that participation was voluntary, responses were anonymous, and data would be used solely for research purposes. Completion and submission of the survey constituted implicit informed consent, as the study posed minimal risk and did not require written or verbal consent.

### 3.3. Analysis method (structural equation model)

The analytical approach used structural equation modeling (SEM) because of its strengths of simultaneously estimating multiple dependency relationships while controlling for measurement error (methodological strengths particularly important for theoretical networks with multiple mediating pathways [16]). Compared to conventional regression techniques, SEM provides more accurate parameter estimates by simultaneously incorporating measurement models into structural model estimation, allowing for a full examination of direct and indirect effects.

The analytical procedure was conducted in two stages following SEM methodological principles: validation of the measurement model in SEM through confirmatory factor analysis (CFA) and estimation of the structural model [52]. This two-stage approach establishes measurement quality before testing structural relationships between constructs and improves the interpretational validity of parameter estimates [13]. Validation of the measurement model incorporated several psychometric criteria: Cronbach's alpha coefficients (threshold >0.7), factor loadings (>0.7), average variance extracted (>0.5), and composite reliability (>0.7) [53]. Assessment of discriminant validity followed the Fornell-Larcker criterion in which the square root of average variance extracted (AVE) for a given construct is greater than its correlations with other constructs.

Estimation of the structural model used maximum likelihood procedures with bootstrapping to generate robust parameter estimates and confidence intervals. Evaluation of model fit examined several goodness-of-fit indices, including chi-square test, comparative fit index (CFI), Tucker-Lewis index (TLI), standardized root mean square residual (SRMR), and root mean square error of approximation (RMSEA). The analytical approach to hypothesis testing incorporated both assessment of direct effects and mediation analysis. Direct effects were assessed through standardized path coefficients, and mediation effects were assessed through bootstrapped indirect effects and associated confidence intervals [17].

Analysis of sequential mediating effects required decomposition of the total indirect effect into specific indirect pathways representing theoretical mechanisms [52]. Several methodological safeguards were also considered, including assessment of common method variance through Harman's single-factor test and a latent method factor approach, examination of alternative model specifications, and sensitivity analysis to assess the stability of parameter estimates under different analytical conditions.

## 4. Data analysis and results

### 4.1. Sample characteristics description

Demographic analysis (Table 2) reveals a gender distribution with slight female predominance (53.0%), while age stratification indicates concentration in younger cohorts (39.0% aged 25–34; 27.4% aged 18–24). Educational attainment metrics demonstrate substantial human capital accumulation (89.4% post-secondary qualifications), suggesting potential correlation between sustainability-oriented festival attendance and higher educational attainment. Festival experience distribution shows predominance of repeat attendees (75.2%), facilitating comparative analysis between novice and experienced participants. Geographical dispersion indicates substantial representation across multiple spatial scales—local (34.4%), regional (38.6%), domestic (19.0%), and international (8.0%)—enabling examination of how spatial proximity influences environmental consciousness and evaluative responses.

**Table 2:** Demographic Profile of Respondents.

Characteristic	Category	Frequency (n)	Percentage (%)
Gender	Male	231	46.2
	Female	265	53
	Non-binary/Other	4	0.8
Age	18-24	137	27.4
	25-34	195	39
	35-44	98	19.6
	45-54	45	9
	55+	25	5
Education	High school or below	53	10.6
	College/University	312	62.4
	Postgraduate	135	27
Prior Music Festival Experience	First-time attendee	124	24.8
	2-3 times	188	37.6
	4-5 times	106	21.2
	More than 5 times	82	16.4
Travel Distance	Local (within 50 km)	172	34.4
	Regional (51–200 km)	193	38.6
	Domestic (>200km)	95	19
	International	40	8
Total		500	100

Note: N = 500.

### 4.2. Measurement model assessment

Given the self-report nature of all measures, common method bias (CMB) was assessed through multiple procedures. Harman's single-factor test revealed the largest unrotated factor accounted for 37.4% of variance, below the 50% threshold indicating severe CMB. Confirmatory factor analysis with a common latent factor (CLF) showed minimal method variance (average CLF loading = 0.09; method factor explained 0.8% variance vs. 68.3% by substantive constructs), with negligible pattern coefficient changes (mean Δ = 0.04). Marker variable analysis (festival merchandising preference, α = 0.82) demonstrated stable correlations after CMV adjustment (average change = 0.03). Collectively, these diagnostics indicated CMB did not substantively compromise measurement validity [49,52,53].

Psychometric assessment began with descriptive statistics (Table 3), revealing correlations ranging from moderate (r = 0.384, ESP-ECP) to strong (r = 0.901, PV-BI). Reliability analysis demonstrated exceptional internal consistency (Cronbach's α = 0.903–0.941; CR = 0.940–0.956). Convergent validity assessment revealed robust item-construct relationships with standardized loadings of 0.888–0.937 and AVE values of 0.810–0.868, substantially exceeding conventional thresholds (Table 4).

**Table 3:** Means, Standard Deviations, and Correlations of the Constructs.

Construct	Mean	SD	1	2	3	4	5	6	7
1. ESP	3.5	0.7	1						
2. SSP	3.48	0.69	0.521**	1					
3. ECP	3.45	0.72	0.384**	0.514**	1				
4. EA	3.47	0.73	0.720**	0.652**	0.564**	1			
5. PV	3.51	0.74	0.712**	0.732**	0.628**	0.817**	1		
6. TS	3.49	0.71	0.705**	0.727**	0.662**	0.756**	0.848**	1	
7. BI	3.5	0.68	0.727**	0.739**	0.665**	0.820**	0.901**	0.872**	1

Note: ESP = Environmental Sustainable Practices; SSP = Social Sustainable Practices; ECP = Economic Sustainable Practices; EA = Environmental Awareness; PV = Perceived Value; TS = Tourist Satisfaction; BI = Behavioral Intentions; ** = Correlation is significant at the 0.01 level (2-tailed).

**Table 4:** Reliability and Validity Assessment of Measurement Model.

Construct	Items	Factor Loadings	Cronbach's α	CR	AVE
Environmental Sustainable Practices (ESP)	ESP1	0.926	0.911	0.944	0.849
	ESP2	0.924			
	ESP3	0.915			
Social Sustainable Practices (SSP)	SSP1	0.909	0.903	0.94	0.839
	SSP2	0.922			
	SSP3	0.916			
Economic Sustainable Practices (ECP)	ECP1	0.93	0.912	0.945	0.851
	ECP2	0.92			
	ECP3	0.918			
Environmental Awareness (EA)	EA1	0.937	0.923	0.952	0.868
	EA2	0.935			
	EA3	0.923			
Perceived Value (PV)	PV1	0.934	0.939	0.956	0.846
	PV2	0.917			
	PV3	0.91			
	PV4	0.918			
Tourist Satisfaction (TS)	TS1	0.926	0.918	0.948	0.859
	TS2	0.927			
	TS3	0.927			
Behavioral Intentions (BI)	BI1	0.893	0.941	0.955	0.81
	BI2	0.916			
	BI3	0.888			
	BI4	0.899			
	BI5	0.903			

Note: CR = Composite Reliability; AVE = Average Variance Extracted.

Discriminant validity evaluation via the Fornell-Larcker criterion (Table 5) revealed generally adequate construct differentiation, with one exception: PV-BI correlation (r = 0.901) marginally approximated √AVE for BI (0.900). Supplementary HTMT analysis confirmed sufficient discriminant validity (PV-BI HTMT = 0.87; all ratios < 0.90). This elevated correlation reflects theoretically coherent linkage wherein value perceptions constitute proximal antecedents of behavioral intentions within value-attitude-behavior hierarchies [34,37,51], rather than conceptual redundancy. Variance inflation factors (range: 1.84–3.47; mean VIF = 2.36) confirmed absence of problematic multicollinearity.

**Table 5:** Discriminant Validity Assessment (Fornell-Larcker Criterion).

Construct	ESP	SSP	ECP	EA	PV	TS	BI
ESP	0.921						
SSP	0.521	0.916					
ECP	0.384	0.514	0.923				
EA	0.72	0.652	0.564	0.932			
PV	0.712	0.732	0.628	0.817	0.92		
TS	0.705	0.727	0.662	0.756	0.848	0.927	
BI	0.727	0.739	0.665	0.82	0.901*	0.872	0.9

Measurement model fit indices (**Table 6**) demonstrated exceptional alignment (CFI = 0.967; TLI = 0.961; RMSEA = 0.046, 90% CI [0.040, 0.052]; SRMR = 0.036; χ²/df = 2.05), establishing robust psychometric foundations for structural hypothesis testing.

**Table 6:** Model Fit Indices for the Measurement and Structural Models.

Fit Indices	Threshold	Measurement Model	Structural Model
Chi-square (χ²)	–	472.68	489.25
Degrees of freedom (df)	–	231	237
χ²/df	<3.00	2.05	2.06
p-value	>0.05	<0.001	<0.001
Comparative Fit Index (CFI)	>0.90	0.967	0.965
Tucker-Lewis Index (TLI)	>0.90	0.961	0.959
Root Mean Square Error of Approximation (RMSEA)	<0.08	0.046	0.046
RMSEA 90% CI	–	[0.040, 0.052]	[0.041, 0.052]
Standardized Root Mean Square Residual (SRMR)	<0.08	0.036	0.048

### 4.3. Structural model validation

**Fig 1:**
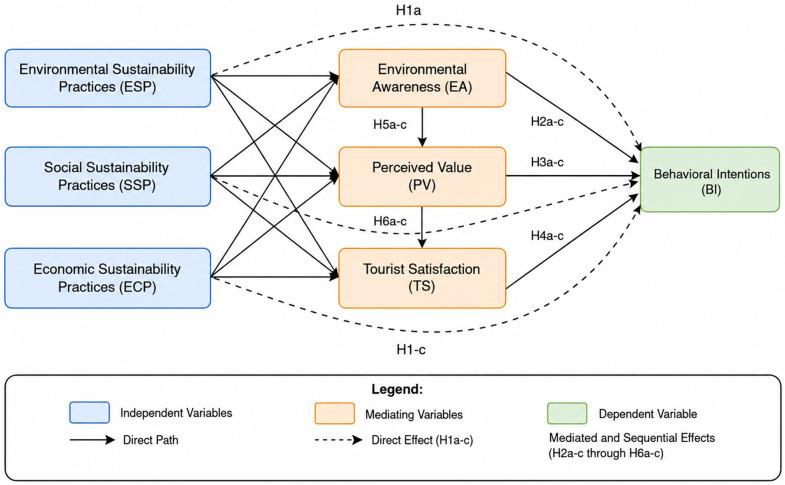
Conceptual Framework of Festival Sustainability Practices and Visitor Behavioral Intentions.

**Fig 2:**
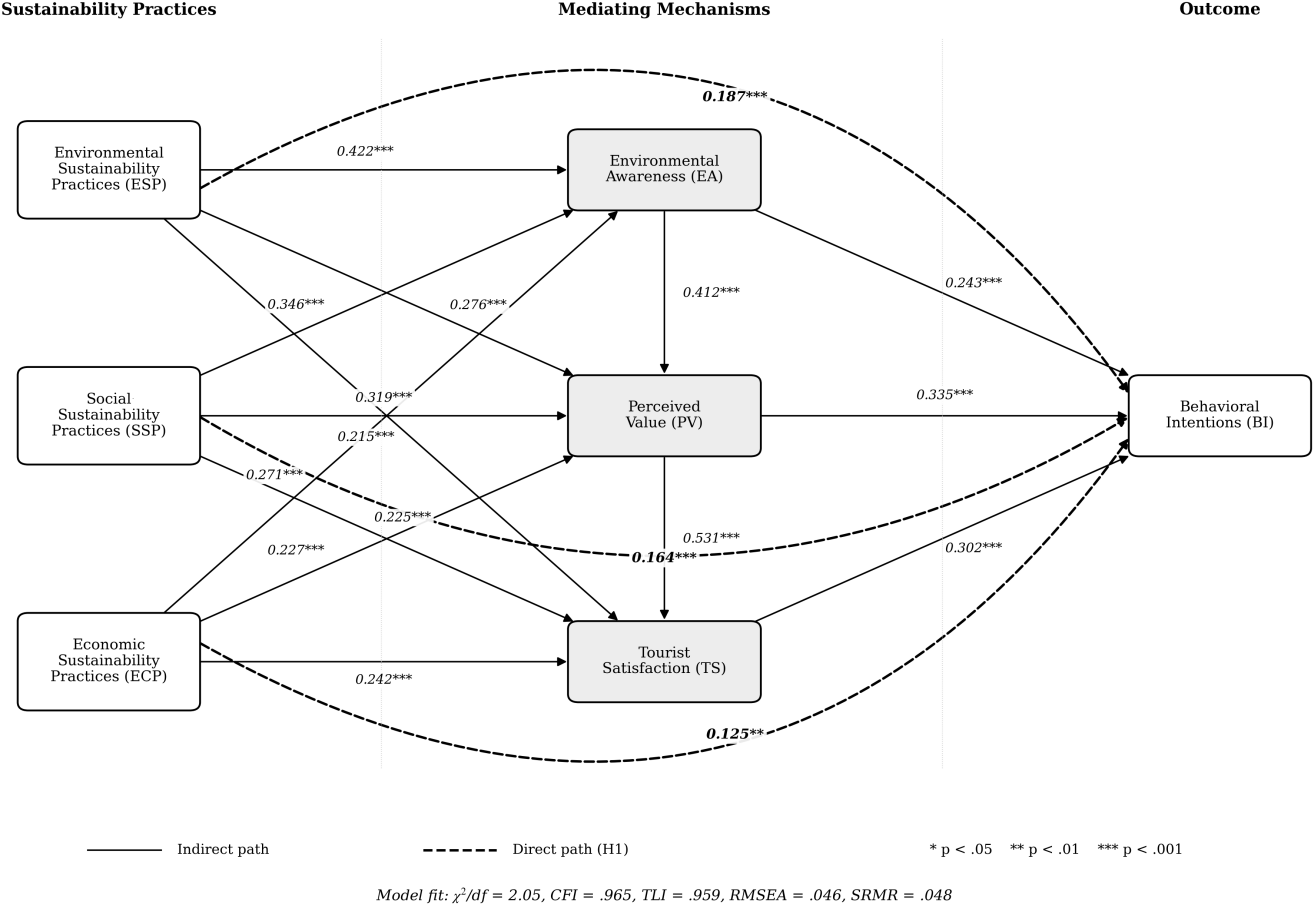
SEM Path Diagram. Note. For visual clarity, observed indicator variables and measurement error terms are omitted from the figure. The full measurement and structural models were estimated simultaneously in the SEM analysis.

The structural model demonstrates exceptional fit to empirical data (CFI = 0.965; TLI = 0.959; RMSEA = 0.046; SRMR = 0.048), with negligible deterioration from measurement model fit indices suggesting reasonable theoretical constraints (Table 6). Direct effects analysis reveals significant path coefficients supporting hypothesized relationships between sustainability practices and behavioral intentions: environmental (β = 0.187, p < 0.001), social (β = 0.164, p < 0.001), and economic (β = 0.125, p = 0.005) sustainability practices all demonstrate significant positive direct effects, supporting hypotheses H1a-H1c respectively (Table 7). All sustainability dimensions demonstrate significant positive effects on hypothesized mediating variables, with standardized coefficients ranging from 0.271 to 0.422 for environmental awareness pathways, 0.227 to 0.319 for perceived value pathways, and 0.215 to 0.242 for satisfaction pathways. Significant relationships between sequential mediating variables further support the theoretical framework's structure (Fig 1),‌‌ with environmental awareness demonstrating significant effect on perceived value (β = 0.412, p < 0.001) and perceived value demonstrating significant effect on satisfaction (β = 0.531, p < 0.001), consistent with the integrated model's emphasis on cascading psychological processes, the SEM Path Diagram is shown in Fig 2.

**Table 7:** Direct Effects in the Structural Model.

Path	Standardized Path Coefficient (β)	t-value	p-value	Hypothesis	Result
ESP → BI	0.187	3.92	<0.001	H1a	Supported
SSP → BI	0.164	3.48	<0.001	H1b	Supported
ECP → BI	0.125	2.79	0.005	H1c	Supported
ESP → EA	0.422	9.38	<0.001	–	–
SSP → EA	0.346	7.64	<0.001	–	–
ECP → EA	0.271	6.15	<0.001	–	–
ESP → PV	0.276	6.44	<0.001	–	–
SSP → PV	0.319	7.52	<0.001	–	–
ECP → PV	0.227	5.48	<0.001	–	–
EA → PV	0.412	8.93	<0.001	–	–
ESP → TS	0.215	4.76	<0.001	–	–
SSP → TS	0.225	5.03	<0.001	–	–
ECP → TS	0.242	5.87	<0.001	–	–
PV → TS	0.531	11.77	<0.001	–	–
EA → BI	0.243	5.08	<0.001	–	–
PV → BI	0.335	5.69	<0.001	–	–
TS → BI	0.302	5.32	<0.001	–	–

Note: ESP = Environmental Sustainable Practices; SSP = Social Sustainable Practices; ECP = Economic Sustainable Practices; EA = Environmental Awareness; PV = Perceived Value; TS = Tourist Satisfaction; BI = Behavioral Intentions.

### 4.4. Mediation effect analysis

Bootstrapped mediation analysis reveals multiple significant indirect pathways through which sustainability practices influence behavioral intentions (Table 8). Environmental awareness demonstrates significant mediating effects between all sustainability dimensions and behavioral intentions, with significant indirect effects for environmental practices (estimate = 0.103, 95% CI [0.061, 0.145]), social practices (estimate = 0.084, 95% CI [0.048, 0.120]), and economic practices (estimate = 0.066, 95% CI [0.037, 0.095]), supporting hypotheses H2a-H2c respectively. Perceived value similarly demonstrates significant mediating effects, with significant indirect effects for environmental practices (estimate = 0.092, 95% CI [0.054, 0.131]), social practices (estimate = 0.107, 95% CI [0.063, 0.151]), and economic practices (estimate = 0.076, 95% CI [0.045, 0.107]), supporting hypotheses H3a-H3c. Tourist satisfaction demonstrates parallel significant mediating effects for environmental (estimate = 0.065, 95% CI [0.036, 0.094]), social (estimate = 0.068, 95% CI [0.038, 0.098]), and economic practices (estimate = 0.073, 95% CI [0.042, 0.104]), supporting hypotheses H4a-H4c.

**Table 8:** Mediation Analysis Results.

Indirect Path	Estimate	95% CI (Lower)	95% CI (Upper)	p-value	Hypothesis	Result
Simple Mediation						
ESP → EA → BI	0.103	0.061	0.145	<0.001	H2a	Supported
SSP → EA → BI	0.084	0.048	0.12	<0.001	H2b	Supported
ECP → EA → BI	0.066	0.037	0.095	<0.001	H2c	Supported
ESP → PV → BI	0.092	0.054	0.131	<0.001	H3a	Supported
SSP → PV → BI	0.107	0.063	0.151	<0.001	H3b	Supported
ECP → PV → BI	0.076	0.045	0.107	<0.001	H3c	Supported
ESP → TS → BI	0.065	0.036	0.094	<0.001	H4a	Supported
SSP → TS → BI	0.068	0.038	0.098	<0.001	H4b	Supported
ECP → TS → BI	0.073	0.042	0.104	<0.001	H4c	Supported
Serial Mediation						
ESP → EA → PV → BI	0.058	0.036	0.08	<0.001	H5a	Supported
SSP → EA → PV → BI	0.048	0.029	0.067	<0.001	H5b	Supported
ECP → EA → PV → BI	0.037	0.022	0.053	<0.001	H5c	Supported
ESP → PV → TS → BI	0.044	0.026	0.062	<0.001	H6a	Supported
SSP → PV → TS → BI	0.051	0.03	0.072	<0.001	H6b	Supported
ECP → PV → TS → BI	0.036	0.021	0.051	<0.001	H6c	Supported

Sequential mediation analysis further reveals significant indirect pathways involving multiple intervening variables. The environmental awareness-perceived value sequential pathway demonstrates significant indirect effects for environmental (estimate = 0.058, 95% CI [0.036, 0.080]), social (estimate = 0.048, 95% CI [0.029, 0.067]), and economic practices (estimate = 0.037, 95% CI [0.022, 0.053]), supporting hypotheses H5a-H5c. The perceived value-satisfaction sequential pathway similarly demonstrates significant indirect effects for environmental (estimate = 0.044, 95% CI [0.026, 0.062]), social (estimate = 0.051, 95% CI [0.030, 0.072]), and economic practices (estimate = 0.036, 95% CI [0.021, 0.051]), supporting hypotheses H6a-H6c.

### 4.5. Hypothesis verification summary

The empirical analysis provides comprehensive support for the proposed theoretical framework, with all eighteen hypotheses receiving statistical confirmation. Direct effect hypotheses (H1a-H1c) receive unequivocal support, with environmental sustainability practices demonstrating strongest direct influence (β = 0.187), followed by social (β = 0.164) and economic dimensions (β = 0.125). Simple mediation hypotheses (H2a-H4c) receive uniform support, with relative magnitudes revealing nuanced patterns: among environmental awareness pathways, environmental sustainability practices demonstrate strongest indirect effect (estimate = 0.103); among perceived value pathways, social sustainability practices demonstrate strongest indirect effect (estimate = 0.107); among satisfaction pathways, economic sustainability practices demonstrate strongest indirect effect (estimate = 0.073). Sequential mediation hypotheses (H5a-H6c) likewise receive uniform support, with environmental sustainability practices demonstrating strongest indirect effect through environment-value pathways (estimate = 0.058) and social sustainability practices demonstrating strongest indirect effect through value-satisfaction pathways (estimate = 0.051).

These findings collectively reveal both the fundamental validity of the theoretical framework and the nuanced patterns of influence across sustainability dimensions and psychological mechanisms. The differential magnitudes across direct and indirect effects elucidate the relative importance of specific sustainability dimensions and psychological pathways in shaping visitors’ behavioral responses, thus contributing to both theoretical advancement and practical management implications.The empirical support for multiple mediation pathways highlights the black box of sustainability’s psychological impact on visitors and how this influences their behavior, which implies that sustainability management should focus not only on practices of implementation but also on their psychological reception and processing by festival attendees.

## 5. Discussion

### 5.1. Research findings and discussion

The empirical analysis elucidates mechanisms through which sustainability practices influence behavioral intentions via direct and mediated pathways. Interpretations adhere to observed statistical evidence while acknowledging measurement limitations and alternative explanatory frameworks.

#### 5.1.1. Direct effects of sustainability practices.

All sustainability dimensions demonstrate significant positive direct effects on behavioral intentions: environmental (β = 0.187, p < 0.001), social (β = 0.164, p < 0.001), and economic (β = 0.125, p = 0.005), supporting H1a-H1c. This differential magnitude extends Son and Lee's festival quality-behavior framework [47] by disaggregating multidimensional sustainability structures. Environmental primacy aligns with documented climate consciousness in contemporary tourism [3], though alternative explanations merit consideration: (1) differential observational visibility—environmental practices (waste reduction, solar power) may be more perceptually salient than social or economic initiatives; (2) sample demographic composition (89.4% post-secondary education) potentially amplifying environmental sensitivity; or (3) measurement specificity differences—environmental items referenced concrete observable practices while economic items addressed abstract constructs (fairness, equity)—potentially generating differential response certainty.

#### 5.1.2. Mediating mechanisms.

Environmental awareness demonstrates significant mediation across all dimensions (H2a-H2c supported; indirect effects: ESP = 0.103, SSP = 0.084, ECP = 0.066), indicating sustainability practices enhance environmental consciousness, subsequently shaping behavioral dispositions. This pattern corroborates Mair and Laing's educational intervention proposition [33], extending beyond attitudinal change to demonstrate awareness-behavior linkages within immersive contexts. However, cross-sectional design limitations preclude definitive temporal inference—heightened awareness may alternatively reflect pre-existing dispositions rather than festival-induced enhancement. Longitudinal designs would better adjudicate between induction versus selection mechanisms.

Perceived value exhibits significant mediation (H3a-H3c supported; indirect effects: ESP = 0.092, SSP = 0.107, ECP = 0.076), with social sustainability demonstrating strongest value-mediated pathway, potentially reflecting cultural resonance whereby community engagement generates identity-congruent perceptions [34,37]. Tourist satisfaction demonstrates parallel mediation (H4a-H4c supported; indirect effects: ESP = 0.065, SSP = 0.068, ECP = 0.073), with economic sustainability exhibiting strongest satisfaction pathway, suggesting instrumental fairness mechanisms contribute substantially to holistic evaluations despite weaker direct effects [41]. Cultural contingencies may moderate these patterns; collectivist orientations in Southeast Asian contexts may amplify economic fairness considerations relative to individualist Western contexts emphasizing environmental symbolism [35].

#### 5.1.3. Sequential mediation pathways.

Sequential mediations through environmental awareness → perceived value → behavioral intentions (H5a-H5c supported; indirect effects: ESP = 0.058, SSP = 0.048, ECP = 0.037) and perceived value → satisfaction → behavioral intentions (H6a-H6c supported; indirect effects: ESP = 0.044, SSP = 0.051, ECP = 0.036) reveal multi-stage psychological processing. These cascading pathways extend VBN theory's linear value-norm-behavior sequences [15] by demonstrating iterative processing stages wherein cognitive appraisals trigger evaluative judgments, subsequently informing affective states culminating in behavioral commitments.

The awareness-value pathway may operate through attention mechanisms: heightened ecological consciousness increases sustainability practice detection, enhancing perceived value through informational processing. Alternative mechanisms include identity-congruence processes, though this interpretation requires direct measurement of environmental self-concept activation not available in current data. The value-satisfaction pathway likely reflects evaluative-affective progression documented in consumer behavior frameworks [37], wherein cognitive value assessments generate subsequent emotional satisfaction states. However, common method variance may partially inflate sequential estimates despite CMB diagnostics indicating minimal bias (method factor = 0.8%).

#### 5.1.4. Construct differentiation: Perceived value and behavioral intentions.

The substantial correlation between perceived value and behavioral intentions (r = 0.901) warrants theoretical interpretation beyond statistical acknowledgment. Discriminant validity diagnostics confirm sufficient construct differentiation (HTMT = 0.87 < 0.90; VIF = 3.47 < 5.0; ΔAVE criterion marginally satisfied), indicating these represent conceptually distinct yet empirically proximate constructs. Within consumer behavior theory, perceived value constitutes evaluative judgment of experiential worth, whereas behavioral intentions encapsulate volitional commitments to future actions [34,51]. Their substantial empirical association reflects genuine psychological proximity wherein value perceptions function as proximal cognitive antecedents of behavioral dispositions, consistent with value-attitude-behavior hierarchical frameworks [37].

Festival contexts may particularly amplify value-intention linkages through: (1) hedonic consumption environments reducing utilitarian calculation complexity, streamlining value-to-action cognitive processing; (2) temporal boundedness concentrating evaluative-behavioral sequences within compressed timeframes; and (3) immersive communal atmospheres facilitating immediate behavioral expression when value perceptions align with perceived normative expectations. Alternative model specifications incorporating second-order constructs or measurement parceling yielded negligible fit improvements (ΔCFI < 0.003), suggesting current two-construct specification adequately captures substantive distinctions without artificial partitioning. Nevertheless, experimental designs with temporal separation of value and intention measurements would strengthen causal inference regarding this theoretically proximate relationship. Conceptually, PV captures an evaluative appraisal of experiential worth—a cognitive judgment formed during or immediately after consumption—whereas BI represents a conative commitment to future action (revisit, recommend, pay premiums). In Fishbein and Ajzen’s attitude–behavior framework, these occupy distinct stages: evaluation precedes volition. Their high empirical correlation in festival settings likely reflects the compressed temporal window of immersive events, where evaluation and intention formation occur nearly simultaneously, amplifying covariance without implying construct redundancy.

### 5.2. Theoretical contributions

This is the first empirically to validate a multidimensional structural model with environmental, social, and economic dimensions building on, yet beyond, environmentally dominant frameworks of being compartmentalized assessments in previous work revealing holistic visitor evaluations in music festivals in line with tendencies of ESG integrations [2,3] but beyond behavioral intentions in post-COVID recovery. By unpacking the “black box” of pro-environmental behavior through mediated understanding of environmental awareness, perceived value, and satisfaction, the model offers a process-oriented lens beyond outcome studies, especially through sequential chains that refine VBN linearity in immersive event contexts [54]. Specifically, whereas VBN theory posits a linear progression from values through beliefs and norms to behavior [15], and MGB extends the Theory of Planned Behavior by incorporating goal-directed desires and past behavior [7], neither framework accounts for the iterative, multi-stage psychological processing that characterizes immersive festival environments. Our sequential mediation findings demonstrate that sustainability practices trigger cascading cognitive–evaluative–affective sequences (awareness → value → intention; value → satisfaction → intention) that are not reducible to VBN’s unidirectional norm activation or MGB’s goal–desire–intention chain. This represents a substantive theoretical advance: the model introduces feedback-compatible, multi-pathway architectures into sustainability–behavior theorizing for event tourism, moving beyond the single-mediator or parallel-mediator designs that have characterized prior applications of these frameworks,these findings resonate with prior work on moral motivation in pro-environmental behavior [55] and the role of psychological factors in green hotel choice [56].

Moreover, by revealing differential pathway effects, such as environmental sustainability dominantly affecting awareness, social influencing value, and economic affecting satisfaction, this study offers dimensional specificity that refines general propositions in event tourism [10] and enables targeted theories for festival contexts beyond holistic treatments in prior models [57]. Our findings support dimensional granularity, and contextual resonances are revealed here; however, future cross-cultural validations may help reduce Western biases in previous models [21]. Methodologically, our application of SEM for assessing multiple effects in one go [13] shows analytical capability in unpacking causal networks beyond generic techniques in unpacking sustainability-specific chains in dynamic events.

Finally, by integrating environmental psychology (e.g., VBN norms), consumer behavior (e.g., value perceptions), and tourism experience models (e.g., satisfaction chains) into one holistic interrelationship, this study breaks silos [12]to enable holistic interrelationships in event sustainability. This integration not only refines MGB's goal processes in festivals but also opens up possibilities for regenerative approaches (e.g., [58]) where future theories may include ESG metrics for more transformative purposes.

### 5.3. Practical implications

The results of this study provide strategic insights for festival organizers, destination managers, and policy makers. For festival organizers, the results require a rethinking of sustainability as no longer being seen as a mere compliance obligation but as a strategic investment. The direct and indirect effects of sustainability practices on behavioral intentions provide empirical support for sustainability to be included in the core business of a festival rather than being seen as peripheral. Differential impact magnitudes show that resources should be optimally allocated to while environmental sustainability influences behavioral intentions most strongly, a balanced approach involving social and economic sustainability may lead to optimal outcomes.

The confirmed mediating function of awareness highlights the need for effective communication strategies. In this regard, festival managers should adopt specific approaches to increase visitor awareness through pre-festival digital communication, interpretive signs and exhibits, interactive demonstrations, and post-event impact studies—educational factors should be embedded in the visitor journey rather than serving as individual information elements. Value enhancement is another important strategic dimension. Sustainability initiatives should be designed to influence multidimensional value perceptions, including not only functional attributes (e.g., the usefulness of transport options) but also emotional attributes (e.g., feelings of being in nature) and social attributes (e.g., self-conceptions of being proenvironmental). Festival designers should adopt systematic value mapping exercises to understand how specific initiatives influence different value dimensions. Given the larger influence of social sustainability initiatives on perceived value, their inclusion is recommended. Drawing on the dimension-specific pathway effects, practitioners can target specific psychological mechanisms: to activate environmental awareness most effectively, organizers should prioritize high-visibility environmental practices such as solar panel installations, real-time waste diversion dashboards, and reusable cup programs (environmental → awareness pathway, β = 0.422); to enhance perceived value, emphasis should be placed on social sustainability initiatives such as local artist showcases, community heritage stages, and accessibility programs (social → value pathway, β = 0.319); and to maximize satisfaction, economic sustainability practices including transparent local sourcing labels, fair-trade vendor partnerships, and economic impact signage should be foregrounded (economic → satisfaction pathway, β = 0.242). This pathway-specific resource allocation framework enables organizers to design sustainability portfolios that simultaneously activate cognitive, evaluative, and affective routes to behavioral loyalty.

For destination managers, sustainability-focused festivals can serve as significant components of broader destination sustainability strategies. The confirmed relationship between festival experiences and environmental awareness highlights how festivals can provide unique opportunities for deep sustainability learning through highly emotionally engaging experiences. For policy stakeholders, our findings provide empirical support for destination sustainability strategies that are based on performance-based regulation aimed at encouraging achievement of sustainability, rather than prescriptive regulations. Given the psychological complexity of sustainability perceptions and responses revealed in this research, implementation strategies should adopt integrated approaches that simultaneously activate multiple pathways rather than isolated initiatives.

### 5.4. Research limitations and future research directions

Although our research makes several contributions, it also has methodological limitations that point to interesting avenues for future research. Our cross-sectional research design limits our ability to draw definitive causal inferences about relationships between sustainability practices and visitor responses [5]. Although our use of structural equation modeling allows for nuanced examination of theoretical relationships, temporal precedence cannot be confirmed without longitudinal data collection. Critically, the sequential mediation pathways reported in this study (e.g., ESP → EA → PV → BI) reflect theoretically hypothesized causal ordering rather than empirically demonstrated temporal sequences; cross-sectional SEM estimates structural associations consistent with the proposed direction but cannot rule out reverse or reciprocal causation. Future research might use multi-wave longitudinal designs to examine how sustainability perceptions develop and change throughout the lifecycle of the festival experience.

Our reliance on self-reported behavioral intentions as the outcome variable rather than observed behaviors may limit our ability to minimize social desirability bias, particularly regarding environmentally-oriented responses [14]. Although self-reported behavioral intentions are well-established proxies for subsequent behaviors in consumer research, the relationship between stated intentions and subsequent behaviors may be moderated by contextual factors. Future research might use objective behavioral measures, such as actual revisitation, documented recommendations, or verified sustainable behaviors, to evaluate the predictive validity of models based on intentions. Moreover, although multiple common method bias diagnostics (Harman’s single-factor test, latent method factor, marker variable analysis) indicated that method variance did not substantively compromise measurement validity, residual common method bias inherent in single-source, single-time-point self-report designs cannot be entirely eliminated. The shared method variance associated with perceptual measures collected simultaneously from the same respondent may partially inflate inter-construct correlations, particularly for theoretically proximate constructs such as perceived value and behavioral intentions. Future studies should consider multi-source designs (e.g., pairing self-reported intentions with observed behavioral data) or temporal separation of predictor and criterion measurements to further mitigate this concern.

Our single-festival research context limits the generalizability of our findings across diverse festival types, geographic contexts, and cultural contexts [59]. Although our selected festival offered appropriate contextual conditions for exploring sustainability perceptions, the relationships between sustainability practices and visitor responses may be moderated by characteristics of different festivals. Future research might use multi-site comparative designs to explore how contextual factors moderate the relationships between sustainability practices and visitor responses in diverse festival environments.

Relatedly, the Southeast Asian festival setting introduces cultural and contextual specificities that warrant explicit discussion. Southeast Asian societies are broadly characterized by collectivist orientations, hierarchical social structures, and community-centered value systems, which may amplify the salience of social sustainability practices (e.g., community engagement, inclusivity) relative to individualist Western contexts where environmental symbolism may dominate visitor evaluations. Furthermore, the region’s rapid economic development trajectory may heighten sensitivity to economic sustainability dimensions such as local vendor support and fair pricing, potentially explaining the relatively strong satisfaction pathway for economic practices observed in this study. The tropical climate and biodiversity-rich settings characteristic of Southeast Asian festivals may also intensify visitors’ environmental awareness responses to sustainability interventions. These cultural contingencies suggest that the relative magnitudes of pathway coefficients reported here may not directly transfer to Western or other non-Asian festival contexts, and future cross-cultural comparative studies are needed to establish boundary conditions for the proposed theoretical model.

Several interesting and substantive dimensions warrant future attention: The potential moderating effects of sociodemographic characteristics on sustainability-behavior relationships; The differential effects of specific sustainability practices beyond general dimensional categories; The potential spillover effects of festival sustainability experiences into visitors’ everyday behaviors; And the emergence of digital and hybrid festival formats during and after the COVID-19 pandemic, which present new sustainability dimensions deserving of special examination of their environmental footprints and visitor perceptions.

In particular, longitudinal panel designs tracking the same visitors across pre-festival, during-festival, and post-festival measurement waves would enable rigorous testing of the temporal ordering assumed by our sequential mediation model, distinguishing festival-induced attitudinal change from self-selection effects. Such designs could also capture whether sustainability-related awareness and value enhancements persist beyond the festival experience or decay upon return to everyday contexts. Additionally, the incorporation of individual-level moderating variables—such as prior festival experience, dispositional environmental orientation (e.g., NEP scores), cultural value dimensions (individualism–collectivism), and sociodemographic characteristics—would clarify for whom and under what conditions sustainability practices most effectively activate the cognitive, evaluative, and affective pathways documented in this study. Multi-group SEM comparisons across these moderator categories would generate actionable segmentation insights for festival managers seeking to tailor sustainability communication strategies to heterogeneous visitor populations.

The pronounced educational homogeneity characterizing our sample constitutes a substantive limitation requiring explicit acknowledgment and theoretical consideration. Demographic analysis reveals 89.4% of respondents possess post-secondary qualifications (62.4% college/university; 27.0% postgraduate), a distributional concentration substantially diverging from general population educational parameters and potentially constraining generalizability across diverse festival attendee populations. Empirical scholarship consistently documents systematic relationships between educational attainment and environmental cognition, with higher education correlating positively with ecological literacy, sustainability discourse familiarity, and pro-environmental behavioral disposition [60]. Within our theoretical framework, this educational skew may amplify observed pathway coefficients through three mechanisms: enhanced cognitive processing capacity for complex sustainability information potentially strengthening environmental awareness mediation; greater facility with multidimensional value constructs potentially intensifying perceived value formation; and more developed future-orientation competencies potentially reinforcing behavioral intention commitment strength. Consequently, relationship magnitudes documented in this investigation may overestimate effect sizes characterizing festivals attracting more educationally heterogeneous attendees or events in contexts with different demographic profiles. Future research should implement stratified sampling designs ensuring proportional representation across educational strata, enabling systematic examination of educational attainment as potential moderating variable through multi-group structural equation modeling. Such investigations would illuminate whether sustainability practice effectiveness varies systematically across educational segments, informing differentiated communication strategies targeting diverse cognitive processing capacities and sustainability knowledge foundations. Additionally, comparative research across festivals with contrasting educational demographic compositions would establish empirical boundaries for theoretical generalization, refining understanding of contextual contingencies moderating sustainability-behavior relationships in event tourism contexts.

## 6. Conclusion

Given the substantive insights gained about the mechanisms through which sustainability initiatives influence visitor responses, this study has systematically examined the complex relationships between music festival sustainability practices and visitors’ behavioral intentions. Study findings support multidimensional sustainability as strategic drivers of intentions, and pathways that support cognitive-evaluative-affective influences—Promoting sustainable development in times of post-COVID [61]. However, overall effects also call for diverse scrutiny [21].

The confirmed direct effects of environmental awareness, perceived value, and satisfaction clarify the complex psychological processes through which sustainability practices lead to behavioral outcomes. These direct mediating effects show that sustainability initiatives influence visitor behavior through multiple cognitive-evaluative-affective pathways that collectively influence future behavioral support. The differential strengths of these effects across sustainability dimensions further show which specific sustainability dimensions influence which specific psychological mechanisms, yielding more substantive understanding of the psychology of sustainability influence in a festival context.

The theoretical contributions make academic debates more profound by offering multidimensional and process understanding of sustainability effects in an event tourism setting. The practical implications offer actionable information to festival organizers and managers, destination managers, and policy stakeholders who wish to achieve both sustainability outcomes and commercial outcomes through integrated approaches -Methodologically, SEM's methodological utility in causal networks expands paradigms like SOR [62]. Practically, guidance promotes integrated approaches, regenerative strategies from community initiatives [63].

Although the research's methodological limitations include methodological issues like cross-sectional design, self-reported measures, and contextual specificity, this research provides strong foundations for further theoretical development through suggested future research directions. Findings together show that effective sustainability management is about more than effective implementation practices, and about the psychological reception and processing by festival attendees of those practices. This visitor-centered perspective goes beyond the typical operational approach to the complex interrelationships between sustainability initiatives and visitors’ experiences and behavioral outcomes in modern festival environments.

## Supporting information

S1 Appendix Complete measurement scales: all questionnaire items, sources, adaptation rationale, and psychometric validation summary. (DOCX)
