# Supplementary material for: Sustainability-induced loyalty in festival tourism: Examining the mediating pathways between sustainable practices and visitor behavioral support
Source: PLoS One. 2026 May 14;21(5):e0348506. doi: 10.1371/journal.pone.0348506 (PMC13175327; doi:10.1371/journal.pone.0348506)
Supplement: S1 Appendix — (DOCX) [file pone.0348506.s001.docx]

**Appendix A: Complete Measurement Scales**

**Measurement Items and Sources**

All items were measured using a 5-point Likert scale (1 = Strongly Disagree, 2 = Disagree, 3 = Neutral, 4 = Agree, 5 = Strongly Agree). Items marked with (adapted) indicate modifications from original scales to enhance contextual relevance for festival environments.

**Environmental Sustainability Practices (ESP)**

**Source:** Adapted from Lück, M. (2003). The New Environmental Paradigm scale in tourism context [27]

**ESP1:** This festival implements visible waste reduction and recycling programs throughout the venue.

**ESP2:** The festival demonstrates commitment to environmental protection through use of renewable energy and resource conservation measures.

**ESP3:** Environmental sustainability is clearly prioritized in the festival's operational practices (e.g., carbon footprint reduction, eco-friendly materials). *(adapted)*

**Adaptation rationale:** Original NEP items were reframed from general environmental beliefs to observable festival-specific environmental practices, maintaining construct validity while enhancing contextual specificity.

**Social Sustainability Practices (SSP)**

**Source:** Adapted from Wong, I.A., Wan, Y.K.P., & Qi, S. (2015). Green events, value perceptions, and consumer involvement [8]

**SSP1:** This festival actively promotes cultural diversity and social inclusivity in its programming and operations.

**SSP2:** The festival demonstrates meaningful engagement with local communities and stakeholders.

**SSP3:** Social equity and accessibility are evident priorities in how this festival is organized. *(adapted)*

**Adaptation rationale:** Wong et al.'s green event attributes were extended beyond environmental focus to encompass social sustainability dimensions, consistent with multidimensional sustainability conceptualizations [3].

**Economic Sustainability Practices (ECP)**

**Source:** Adapted from Jones, M. (2017). Sustainable Event Management framework [3]

**ECP1:** This festival prioritizes local vendors and suppliers in its economic operations.

**ECP2:** The festival demonstrates transparent and fair pricing practices.

**ECP3:** Economic benefits from this festival appear to be distributed equitably among stakeholders. *(adapted)*

**Adaptation rationale:** Items operationalize economic sustainability as observable festival practices rather than abstract principles, enhancing measurement validity in visitor perception contexts.

**Environmental Awareness (EA)**

**Source:** Adapted from Üzülmez, M., Ercan İştin, A., & Barakazı, E. (2023). Environmental awareness in tourism contexts [28]

**EA1:** Attending this festival has increased my awareness of environmental issues.

**EA2:** This festival experience has made me more conscious about environmental protection.

**EA3:** I have gained greater understanding of sustainable practices through participating in this festival. *(adapted)*

**Adaptation rationale:** Domain-specific environmental awareness items were contextualized to capture festival-induced consciousness enhancement, reflecting temporary community learning dynamics [33].

**Perceived Value (PV)**

**Source:** Adapted from Sánchez, J., Callarisa, L., Rodríguez, R.M., & Moliner, M.A. (2006). Multidimensional perceived value framework [34]

**PV1:** Considering what I paid, this festival offers excellent value for money. *(Functional value)*

**PV2:** Attending this festival makes me feel good and gives me pleasure. *(Emotional value)*

**PV3:** This festival experience contributes positively to my self-image and how others perceive me. *(Social value)*

**PV4:** The sustainability practices at this festival enhance the overall value I receive from my experience. *(adapted - Sustainability-enhanced value)*

**Adaptation rationale:** Sánchez et al.'s functional-emotional-social value dimensions were retained, with PV4 added to capture sustainability-specific value perceptions central to this study's theoretical framework.

**Tourist Satisfaction (TS)**

**Source:** Adapted from Thrane, C. (2002). Festival satisfaction measurement [50]

**TS1:** Overall, I am very satisfied with my experience at this festival.

**TS2:** This festival has met my expectations.

**TS3:** My decision to attend this festival was a wise one. *(adapted)*

**Adaptation rationale:** Minimal adaptation from Thrane's festival-validated items, with TS3 reframed to emphasize decision validation consistent with post-consumption evaluation frameworks [37].

**Behavioral Intentions (BI)**

**Source:** Adapted from Tkaczynski, A., & Stokes, R. (2010). FESTPERF scale [51]

**BI1:** I intend to revisit this festival in the future.

**BI2:** I will recommend this festival to friends and family.

**BI3:** I am willing to pay a premium price for a festival with strong sustainability practices. *(adapted)*

**BI4:** I will speak positively about this festival to others.

**BI5:** I intend to continue supporting festivals that prioritize sustainability. *(adapted)*

**Adaptation rationale:** FESTPERF revisit and recommendation items (BI1, BI2, BI4) were supplemented with sustainability-specific behavioral intentions (BI3, BI5) to capture willingness-to-pay and sustained support—outcome variables theoretically central to sustainability-behavior relationships [7, 14].

**Psychometric Validation Summary**

Expert panel review (n=5 academics; n=3 practitioners) confirmed content validity (CVI > 0.85 for all items). Pilot testing (n=90) demonstrated acceptable reliability (Cronbach's α range: 0.84-0.91) and no systematic comprehension difficulties. Full sample confirmatory factor analysis (N=500) yielded robust psychometric properties reported in Table 4 (factor loadings: 0.888-0.937; Cronbach's α: 0.903-0.941; CR: 0.940-0.956; AVE: 0.810-0.868), confirming measurement validity and reliability.
